# Comparative Transcriptome Analysis Reveals Different Responses in Three Developmental Stages of *Mythimna loreyi* to Cold Stress

**DOI:** 10.3390/insects15070554

**Published:** 2024-07-22

**Authors:** Yun Duan, Qi Chen, Muhammad Bilal, Yuqing Wu, Zhongjun Gong, Renhai Wu, Jin Miao

**Affiliations:** 1 Henan Key Laboratory of Crop Pest Control, Key Laboratory of Integrated Pest Management on Crops in Southern Region of North China, Institute of Plant Protection, Henan Academy of Agricultural Sciences, Zhengzhou 450002, China; duanyunhao@163.com (Y.D.); bilalsaif987@gmail.com (M.B.); yuqingwu36@hotmail.com (Y.W.); gongzj_2@hotmail.com (Z.G.); 2 Luohe Academy of Agricultural Sciences, Luohe 462000, China; chenqi9992@sina.com; 3 College of Plant Protection, Henan Agricultural University, Zhengzhou 450002, China

**Keywords:** *Mythimna loreyi*, transcriptome, cold stress, developmental stage

## Abstract

**Simple Summary:**

Simple Summary: The loreyi leafworm, *Mythimna loreyi* (Duponchel), has recently expanded its distribution range to many countries around the world. To understand the molecular response mechanism of this pest to cold stress, a comprehensive transcriptome analysis was carried out in three developmental stages (larvae, pupae, and adults) of *M. loreyi*. The results indicated that there were significant differences in metabolism-related processes and gene expression when they were exposed to low temperatures. In addition, several differentially expressed genes were identified in these stages after exposure to cold stress. This is the first study to test cold stress-induced transcriptional changes in three developmental stages of *M. loreyi* and provide basic data for understanding the molecular response mechanisms of moths under cold stress.

**Abstract:**

The loreyi leafworm *Mythimna loreyi* (Lepidoptera: Noctuidae) is a serious pest of agriculture that causes particular damage to Gramineae crops in Asia, Europe, Australia, Africa, and the Middle East. Low temperature is one of the important environmental factors that limits the survival, distribution, colonization, and abundance of *M. loreyi*. However, the metabolic synthesis pathways of cold-tolerant substances in *M. loreyi* and the key genes involved in the regulation under cold stress remain largely unknown. In this study, we sequenced the transcriptomes of three developmental stages (larvae, pupae, and adults) of *M. loreyi* to discover the molecular mechanisms of their responses to cold stress. In total, sequencing generated 120.64 GB of clean data from 18 samples, of which 19,459 genes and 1740 differentially expressed genes (DEGs) were identified. The enrichment analysis of Gene Ontology (GO) and Kyoto Encyclopedia of Genes and Genomes (KEGG) revealed that many DEGs were mainly enriched in pathways associated with energy metabolism and hormone metabolism. Among these, genes encoding multiple metabolic enzymes, cuticle proteins (CPs), and heat shock proteins (HSPs) were differentially expressed. These results indicate that there are significant differences among the three developmental stages of *M. loreyi* exposed to cold stress and provide a basis for further studying the molecular mechanisms of cold tolerance in insects.

## 1. Introduction

The loreyi leafworm *Mythimna loreyi* (Duponchel) (Lepidoptera: Noctuidae) is a serious and polyphagous pest for agriculture that damages many grain crops and grasses from the Gramineae family, including wheat, maize, rice, barley, sugarcane, and others [1,2]. It is widely dispersed in many countries in Asia, Europe, Australia, Africa, and the Middle East [3,4], and it has enormous destructive potential for agriculture. Recently, *M. loreyi* has increased worldwide and has become a common pest in many countries and regions [4,5,6]. In China, *M. loreyi,* typically occurring together with *Mythimna separata*, has caused a great threat to agricultural production, and its damage levels continue to increase [7]. In 2020 and 2023, *M. loreyi* was listed on the list of first-class crop pests by the Ministry of Agriculture and Rural Areas of China.

*M. seperata* and *M. loreyi* are closely related species [8]. They undergo multiple generations per year and overwinter most likely as larvae or pupae in the soil. The overwintering stages of these armyworms are more tolerant to low temperatures than non-overwintering stages; this is essential to their successful winter survival and population maintenance. The population density of armyworms in the subsequent year is determined by their overwintering survival rate, which is largely dependent on temperature. Previous studies showed that *M. separata* had weak resistance to cold stress and the 0 °C isotherm of the minimum mean temperature of January was proposed as its overwintering range limit in eastern China [9]. In recent years, the overwintering range of *M. loreyi* has expanded in north China with current trends in global warming [5]. At present, research on the ability of this species to withstand cold has been mostly focused on super-cooling points (SCPs) and freezing points (FPs). Qin et al. [10] found significant differences between cold hardiness and SCPs across different developmental stages of *M. loreyi*. Five-day-old pupae with an average SCP of −16.69 °C were considered as the most cold-tolerant life stage, followed by three-day-old pupae (−16.20 °C). In the laboratory, the lower threshold temperatures of the larval, pupal, and adult period of *M. loreyi* were estimated to be 10.95, 11.67, and 9.65 °C, respectively [11]. Although the temperature effects on the ecological performances of *M. loreyi* at different stages have been extensively investigated, the mechanisms of their response to low temperature have not been studied, and the key genes and pathways involved in the regulation remain largely unknown.

Insects are temperature-sensitive animals and can adjust their physiology and behavior according to changes in ambient temperature [12,13,14]. Low temperature is among the important environmental elements affecting the survival, distribution, colonization, abundance, behavior, and life history of insects [15,16,17]. To resist cold stress and maintain the population, insects have formed a series of strategies to improve their response-ability to withstand low-temperature changes in the long-term evolution [18,19]. When subjected to low temperature, insects are first regulated at the gene level and transcription level, and then they are controlled by metabolic synthesis to cope with the damage caused by cold stress [20,21]. Therefore, to fully comprehend the mechanisms of insect resistance to cold, a detailed investigation of the genetic and metabolic changes they undergo in response to cold stress is essential.

In recent years, given the quick growth of high-throughput sequencing technology, transcriptome sequencing (RNA-seq) has been widely used for studying how insects react to cold stress at the molecular level and other abiotic stresses [22,23]. Previous studies indicated that multiple differentially expressed genes (DEGs) associated with cold stress, such as cuticle proteins (*CPs*), heat shock proteins (*HSPs*), glucose dehydrogenase (*GLD*), and hormone-related proteins, were identified in insects, including *Galeruca daurica* [24], *Plutella xylostella* [25], *Anoplophora glabripennis* [26], and *Liriomyza trifolii* [27]. Comparing the transcriptomes of *Hyles euphorbiae* under low temperatures found 605 differentially expressed transcripts, and those genes coding for detoxification enzymes were upregulated [28]. Enriquez and Colinet [29] found that metabolic pathways, including purine metabolism and aminoacyl tRNA biosynthesis, were involved in female *Drosophila suzukii* in response to cold stress. Furthermore, Zhang et al. [30] discovered that cold tolerance in *G. daurica* was associated with the pathways for fatty acid biosynthesis, heat shock protein synthesis, and glycolysis/gluconeogenesis. These studies provide insights into how transcriptional changes occur during insect response to cold stress. Additionally, many studies revealed that cold hardiness can vary substantially within a species, both among and within individuals and different developmental stages [31]. However, information related to physiological and molecular mechanisms in insect response to cold stress is currently limited. While a great deal of focus has been placed on only one stage within a species [22,24], the consequences of mild cold conditions have not been thoroughly studied in different developmental stages of insects. The current investigation sought to understand how *M. loreyi* responds to mild cold stress (5 °C) and whether and how transcriptome responses to such stress can vary between stages within a single species. In this study, three developmental stages (larvae, pupae, and adults) of *M. loreyi* were kept at room temperature (25 °C) as a control and cold shock. Transcriptome sequencing was employed to evaluate and describe the expression of genes, as well as to identify genes and pathways that might be involved in their cold tolerance. Our results illustrate the possible mechanisms of cold tolerance and overwintering capacities in different stages of *M. loreyi*. These data can offer fresh insights into how nondiapausing insects withstand cold temperatures and aid in our investigation into the significance of temperature restrictions to this highly mobile pest species.

## 2. Materials and Methods

### 2.1. Insect Rearing and Low-Temperature Treatments

A colony of *M. loreyi* derived from a field collection near Luoyang, Henan province, China (34°58′ N, 111°73′ E; 83 m), was reared on maize under laboratory conditions at 25 ± 1 °C and 70 ± 5% relative humidity, with a 14 L:10 D photoperiod. Pupae had separate housing and were sexed until eclosion. For cold stress treatment, third instar larvae (*n* = 10/sample), three-day-old pupae (1 female and 1 male), and three-day-old adults (1 female and 1 male) were collected in 50 mL disposable lunch boxes and incubated at 5 °C for 3 h in a cryogenic incubator (DKCG150, Ningbo Prand Instrument Co., Ltd., Ningbo, China) and then recovered at 25 °C for 1 h as treatment groups. After cold stress treatment, the larvae, pupae, and adults were active and no mortality appeared. Therefore, all the individuals from different treatments were collected as samples for transcriptome sequencing. Each treatment consisted of three replicates. Meanwhile, the same developmental stages of *M. loreyi* under rearing conditions (25 °C) were collected for control groups. A total of 18 samples (9 samples from treatment groups and 9 from control groups) were used for the final transcriptome sequencing, and before being extracted for RNA, the samples were flash-frozen in liquid nitrogen and maintained at −80 °C.

### 2.2. RNA Extraction, cDNA Library Construction, and Sequencing

Total RNA from 18 samples was extracted according to the instructions of TRIzol reagent (Invitrogen, CarIsbad, CA, USA). RNA purity and RNA integrity were assessed with a NanoPhotometer^®^ spectrophotometer (IMPLEN, CA, USA) and an RNA Nano 6000 Assay Kit of the Agilent Bioanalyzer 2100 system (Agilent Technologies, Santa Clara, CA, USA), respectively. Out of all the RNA samples, eighteen libraries were produced for Illumina RNA sequencing.

The mRNA was purified from total RNA using magnetic beads attached to poly-T oligo. Next, using divalent cations at a high temperature, the enriched mRNA was broken up into short pieces in NEBNext First Strand Synthesis Reaction Buffer (5×). A random hexamer primer and M-MuLV Reverse were used to make first-strand cDNA, and second-strand cDNA synthesis was subsequently performed using DNA Polymerase I and RNase H. The library fragments were purified using AMPure XP (Beckman Coulter, Beverly, MA, USA) to choose cDNA fragments that were preferably 250–300 bp in length. After that, adaptor-ligated, size-selected cDNA was used with 3 μL USER Enzyme (NEB, Ipswich, MA, USA) at 37 °C for 15 min, and then 5 min at 95 °C, before PCR. PCR was conducted using Phusion High-Fidelity DNA polymerase, universal PCR primers, and indes (X) primer. After the PCR products were purified using the AMPure XP system, the Agilent Bioanalyzer 2100 system (Agilent Technologies, Santa Clara, CA, USA) was utilized to assess the quality of the libraries. The Illumina RNA sequencing was performed by Novogene Bioinformatic Technology Company Limited (Beijing, China). Paired-end sequencing with a 150 bp read length was performed on the HiSeqTM2000 platform from Illumina.

### 2.3. RNA-Seq Analysis of Data

To create clean reads, the adaptor, poly-N, and low-quality reads were removed from the raw data using the Fast v 0.20.0 software (https://github.com/OpenGene/fastp, accessed on 10 April 2019). Concurrent calculations were made for the Q20, Q30, and GC contents of the clean data. Clean, high-quality data served as the foundation for all downstream studies.

Next, next-generation sequencing reads were mapped, distributed, and counted across genes and transcripts to perform RNA-seq analysis. As a reference genome, the most recent assembly of the *M. loreyi* genome (NCBI Assembly: GCA_029852875.1) was employed. Following quality assurance sequencing, the reads were compared to a reference genome. Clean readings were promptly and precisely compared to a reference genome by using HISAT2 (v2.0.5) (http://daehwankimlab.github.io/hisat2, accessed on 5 November 2016). Gene expression abundance was assessed using FPKM (fragments per kilobase of transcript sequence per million base pairs sequenced) values [32], which were obtained by comparing the location information of the gene to the reference genome of *M. loreyi*. Statistical and quantitative analysis of the number of reads covered by each gene was carried out using feature Counts (1.5.0-p3).

### 2.4. Differential Expression Gene Analysis

Normalization and statistical model hypothesis testing probability (*p*-value) were carried out on the initial read count following the quantification of gene expression. To adjust the *p*-values, multiple hypothesis testing was carried out [33,34]. The assembled transcriptomes of cold-stressed samples were compared with control samples. Differential expression analysis for treatments and controls was carried out using the DESeq2 R package (1.20.0) [35], with the thresholds for significantly differential expression being set at | log2 fold change | > 0.5 and FDR (false discovery rate) < 0.05. Venn diagrams were created using Venny 2.1.0 (BioinfoGP Service, Centro Nacional de Biotecnología (CNB-CSIC), Madrid, Spain).

### 2.5. GO and KEGG Enrichment Analysis of DEGs

Gene Ontology (GO, http://geneontology.org/) (accessed on 3 November 2023) and the Kyoto Encyclopaedia of Genes and Genomes (KEGG) (https://www.genome.jp/kegg/, accessed on 20 April 2023) enrichment analysis was used to determine the gene functions and associated biochemical and signal transduction pathways of DEGs. The cluster profile R package was used, with a cut-off of *p*-value < 0.05, in which gene length bias was corrected. The findings of the GO analysis were categorized into three distinct hierarchies: “Biological Process” (BP), “Cellular Component” (CC), and “Molecular Function” (MF).

### 2.6. Validation of RNA-Seq Analysis by qRT-PCR

To confirm the transcriptome data, four DEGs from larvae (PYW08_008574, PYW08_000756, PYW08_011180, and PYW08_007365), pupae (PYW08_014508, PYW08_002107, PYW08_008323, and PYW08_010826), and adults (PYW08_010836, PYW08_010880, PYW08_005434, and PYW08_015979) of *M. loreyi* were chosen randomly for the qRT-PCR test, and translation elongation factor 2 (*EF2*) was used as a reference gene. Primer Premier 5.0 was used to create the primers (Table S1).

All samples for the qRT-PCR test were treated in the same way as the sequenced samples. Total RNA from each sample was extracted using RNAiso Plus (Takara, Dalian, China) based on the manufacturer’s instructions. After that, the genomic DNA contamination was eliminated from the total RNAs by treating them with DNase I (TaKaRa, Dalian, China). Using a PrimeScriptTM RT reagent Kit with a gDNA Eraser (Takara, Dalian, China), 1 μg of total RNA was converted into first-strand cDNA. As stated by Liu et al. [36], qRT-PCR was carried out in 20 μL reaction volumes utilizing a Thermal Cycler Dice Real Time System Lite (TaKaRa, Dalian, China). Every experiment had negative controls that lacked transcriptase or a template. Using the 2^−ΔΔCt^ technique, the relative expression levels of DEGs were examined from three separate biological replicates [37], and the data were shown as means ± standard errors.

## 3. Results

### 3.1. Sequencing, Assembly, and Transcriptome Functional Annotation

To study possible molecular mechanisms in the different developmental stages of *M. loreyi* under cold stress, 18 cDNA libraries from larvae, pupae, and adults that had been exposed to 5 °C (treatment samples) (ML_Lt1, ML_Lt2, ML_Lt3, ML_Pt1, ML_Pt2, ML_Pt3, ML_At1, ML_At2, and ML_At3) and from the same developmental stages under 25 °C (control samples) (ML_Lck1, ML_Lck2, ML_Lck3, ML_Pck1, ML_Pck2, ML_Pck3, ML_Ack1, ML_Ack2, and ML_Ack3) were prepared for illumine sequencing. The transcriptome sequencing data created during this investigation have been added to the Short Read Archive at NCBI (SRA) linked to Bio Project: PRJNA1096267.

Totally, 122.89 GB of raw reads were yielded from 18 libraries. Subsequently, a rigorous quality control study was conducted on every sample to confirm the authenticity of these sequencing data. Following the removal of the unclear nucleotides and poor-quality sequences, 120.64 GB of clean reads (98.17% of raw reads) were generated. Furthermore, 255.65, 274.50, and 274.15 million uncontaminated readings were obtained from larvae, pupae, and adults, respectively. For every sample, the number of clean bases varied from 6.15 to 7.33 G. The averages of Q20, Q30, and GC content of clean reads were 98.13%, 94.40%, and 46.38%, respectively (Table 1).

Afterward, these clean reads were mapped to the *M. loreyi* genome, and the mapped rates ranged from 84.22% to 87.88% for 18 samples (Table S2). A total of 80.62–85.24 percent of the readings could be uniquely mapped. Nearly all of the read 1, read 2, positive, and negative reads that were mapped to the genome were higher than 40.07%. The range of read pairs that were mapped to the genome was 73.21–79.17%. A total of 16,970 genes, or 87.21% of the total anticipated genes, could be effectively annotated in the *M. loreyi* genome out of the 19,459 genes that were discovered from all the samples combined. The 2489 genes that remained unannotated in the *M. loreyi* genome were classified as new genes.

### 3.2. Analysis of Differentially Expressed Genes

**Figure 1:**
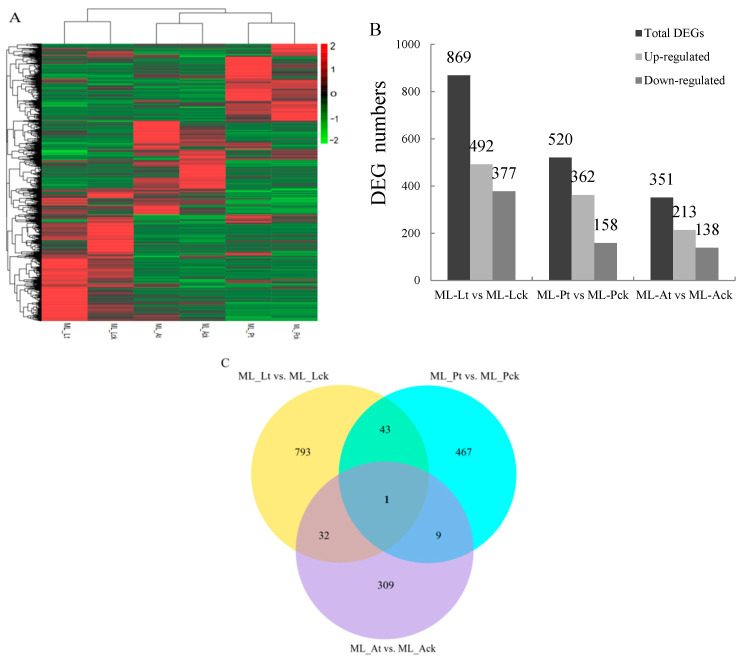
Differentially expressed genes (DEGs) in the larvae, pupae, and adults of *M. loreyi* exposed to cold stress. (**A**) Expression profiles (FPKM value) of DEGs. *X*-axis: sample name, *Y*-axis: DEGs. The colors indicated the expression levels of DEGs, and red indicated high expression and green indicated low expression. ML-Lt, ML-Pt, and ML-At represent larvae, pupae and adults exposed to cold stress, respectively; ML-Lck, ML-Pck, and ML-Ack represent larvae, pupae and adults under control treatments, respectively. (**B**) The number of differentially expressed genes (DEGs) in larvae, pupae or adults of *M. loreyi* under cold stresses. All values were obtained based on discriminative power (|fold change| ≥ 0.5 and FDR ≤ 0.05). (**C**) Venn diagram of DEGs associated with cold stress in larvae, pupae and adults of *M. loreyi* compared to control.

To gain insight into the transcriptomic profiles of the response of *M. loreyi* to cold stress, genes expressed in the larvae, pupae, and adults were compared between the cold stress and control treatments. Between cold stress and the control (| fold change | > 0.5 and FDR ≤ 0.05), a total of 1740 differentially expressed genes (DEGs) were found (Figure 1A). Of these, 869, 520, and 351 DEGs were found in larvae, pupae, and adults, respectively (Figure 1B; Tables S3–S5). Among these, 492, 362, and 213 genes were upregulated in larvae, pupae, and adults, respectively, while 377, 158, and 138 genes were downregulated in the corresponding stages. Venn analysis showed that there were 44, 33, and 9 common DEGs between the comparison sets of larvae and pupae, larvae and adults, and pupae and adults, respectively (Figure 1C).

### 3.3. Gene Functional Annotations of DEGs

To assess if *M. loreyi* proteins of these DEGs are related to any significant biological relevance, GO analysis was conducted using up- and downregulated genes. According to the GO classification, approximately 31.08–34.69% of these DEGs were distributed in BP, 48.07–51.89% in MF, and only 16.33–17.86% in CC in three stages of *M. loreyi* (Table 2).

The top 10 statistics of the GO analysis of these DEGs are listed in Figure S1. The results showed that defense response was simultaneously enriched in the top 10 in BP in three developmental stages. Response to external stimulus, response to biotic stimulus, response to external biotic stimulus, response to other organisms, and defense response to other organisms were simultaneously enriched in the top 10 in BP in pupae and adults. Cofactor binding and coenzyme binding were simultaneously enriched in MF in larvae and pupae. Additionally, non-membrane-bounded organelles were simultaneously enriched in the top 10 in CC in larvae and adults.

GO enrichment analysis was carried out to look more closely into the roles of these DEGs. A total of 10, 3, and 1 significantly enriched GO terms with a *p*-value < 0.05 were identified in larvae, pupae, and adults, respectively (Table 3), including cofactor binding, flavin adenine dinucleotide binding, coenzyme binding, iron-sulfur cluster binding, metal cluster binding, oxidoreductase activity, and electron transfer activity in the larvae, and vitamin binding, pyridoxal phosphate binding, and vitamin B6 binding in the pupae. Only odorant binding was substantially enriched in adulthood.

### 3.4. KEGG Enrichment Analysis of DEGs

To investigate the enriched metabolic pathways or signal transduction pathways of DEGs with a *p*-value < 0.05, KEGG enrichment analysis was also carried out. In total, 184, 97, and 47 DEGs were enriched in 99, 79, and 53 KEGG pathways in larvae, pupae, and adults, respectively. The top 20 pathways of DEGs are shown in Table 4. The findings demonstrated that DEGs in larvae were significantly enriched in the following pathways: peroxisome, metabolism of xenobiotics by cytochrome P450, biosynthesis of cofactors, pentose, and glucuronate interconversions. In pupae, the longevity regulating pathway of multiple species and protein processing in the endoplasmic reticulum were highly enriched, while in adults, no significantly enriched pathway was detected.

### 3.5. Changes in the Expression Level of Genes Encoding Metabolic Enzymes

**Figure 2:**
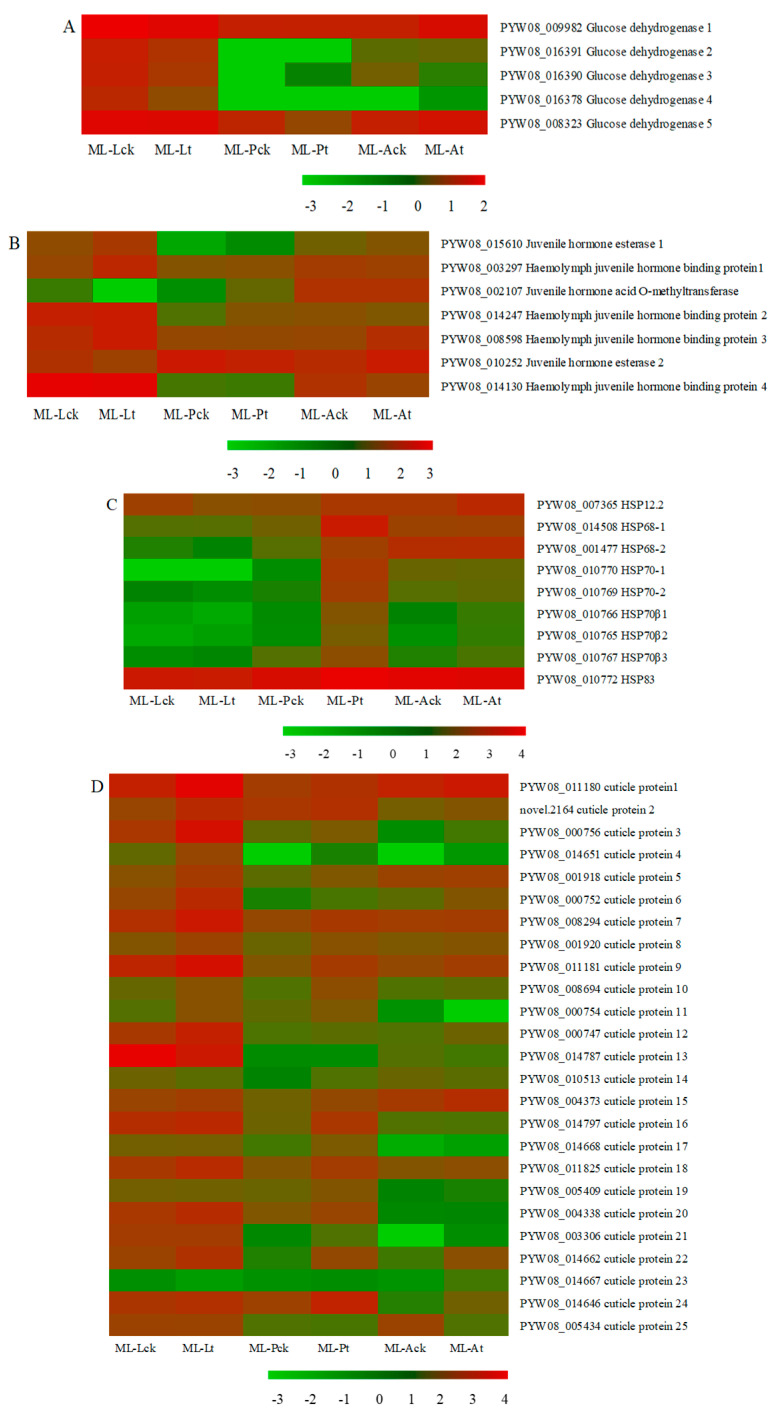
Expression profiles (FPKM value) of the DEGs. *X*-axis: sample name, *Y*-axis: gene name. The colors indicated the expression levels of genes, and red indicated high expression and green indicated low expression. (**A**) glucose dehydrogenase (*GLDs*); (**B**) genes involved in JH signaling and metabolism; (**C**) heat shock proteins (*HSPs*); (**D**) cuticle proteins (*CPs*).

Previous studies revealed that many metabolic processes could be regulated in insects under cold stress, including the metabolism of lipids, carbohydrates, and amino acids [29,30]. In this study, GO and KEGG enrichment analyses of DEGs indicated that many metabolic enzymes were differentially expressed in *M. loreyi* in three stages after exposure to 5 °C (Tables S3–S5). Among these metabolisms, glucose metabolism was very notable. In total, five genes encoding glucose dehydrogenase (*GLD*) were differently expressed in larvae, pupae, and adults after exposure to cold stress (Figure 2A). Among these *GLD* DEGs, three genes (PYW08_016391, PYW08_016390, and PYW08_016378) were highly downregulated in larvae and one gene (PYW08_008323) was downregulated in pupae. Additionally, one gene (PYW08_009982) was downregulated in larvae but upregulated in adults.

Stress conditions had a significant impact on hormone biosynthesis, metabolism, and signaling, which were then regulated by insect tolerance to stresses. In this study, seven DEGs involved in juvenile hormone (JH) signaling and metabolism were differentially expressed in three stages of *M. loreyi* under low-temperature stress (Figure 2B). These DEGs included two upregulated genes (PYW08_015610 and PYW08_010252) encoding juvenile hormone esterase (*JHE*), one upregulated gene (PYW08_002107) encoding juvenile hormone acid O-methyltransferase (*JHOM*), and four genes (three upregulated (PYW08_003297, PYW08_014247, and PYW08_008598) and one downregulated (PYW08_014130)) encoding hemolymph juvenile hormone binding protein (*JHBP*).

In addition, many metabolic enzymes involved in sugar, lipid, and amino acid metabolisms were also identified in this study. For example, genes encoding UDP-N-acetylhexosamine pyrophosphorylase-like protein, UDP-glucoronosyl, UDP-glucosyl transferase, phosphatidate phosphatase, ecdysteroid UDP-glucosyltransferas, and fatty acyl-CoA reductase were dramatically upregulated in larvae after exposure to cold stress. In pupae, the most highly upregulated genes included fatty acyl-CoA reductase and UDP-glucosyltransferase. Meanwhile, two genes encoding fatty acid synthase (*FAS*) were highly downregulated in *M. loreyi* larvae and adults compared to the control.

### 3.6. Changes in the Expression Level of HSPs Genes

The transcriptional response of insects to cold stress always includes a number of HSPs, suggesting a universal mechanism of insect cold tolerance. In this study, nine *HSP* genes were differentially expressed, including one, two, three, two, and one of the subfamilies of *HSP12.2*, *HSP70*, *HSP70b*, *HSP68*, and *HSP83*, respectively (Figure 2C). Among these DEGs of *HSPs*, eight genes were remarkably upregulated in pupae under cold stress, and seven of these eight genes had a greater than three-fold increase in expression. In addition, one *HSP12.1* was upregulated in adults but highly downregulated in larvae. These results indicated that HSPs might have a vital role in cold tolerance in *M. loreyi*.

### 3.7. Changes in the Expression Level of CP Genes

Insect cuticles are made up primarily of cuticle proteins (CPs), which are crucial for the way insects react to cold stress. Our findings showed that when *M. loreyi* was exposed to cold stress, the expression of 25 genes (22 upregulated and 3 downregulated) encoding CPs varied. These genes included 14 *CPs* (12 upregulated and 2 downregulated) in larvae, 11 upregulated *CPs* in pupae, and 5 *CPs* (4 upregulated and 1 downregulated) in adults (Figure 2D). Of these DEGs of *CPs*, one was commonly upregulated in larvae and adults (PYW08 000752) and four were commonly upregulated in larvae and pupae (PYW08 0001918, PYW08 0008294, PYW08 011181, and PYW08 008694). These findings suggested that cuticle proteins, which are typically elevated, are important components of *M. loreyi* response to low temperatures.

### 3.8. Validation of RNA-Seq Analysis by qRT-PCR

**Figure 3:**
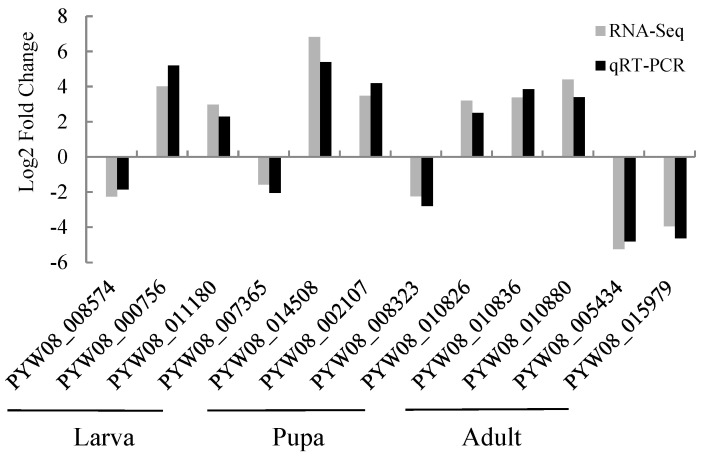
qRT-PCR results of 12 DEGs.

By using qRT-PCR, the expression of 12 DEGs was examined in cold-stressed larvae, pupae, and adults of *M. loreyi* to confirm the validity of RNA-seq findings. The credibility of the DEG data is confirmed by the qRT-PCR analysis results, which were in line with the expression of 12 DEGs in the RNA-seq data (Figure 3).

## 4. Discussion

*M. loreyi* is a migratory species and an important insect pest of Gramineae crops worldwide. Current studies indicated that *M. loreyi* has expanded its northern distribution in many countries in the world due to a warming climate [4,6,38]. However, the molecular mechanisms underlying *M. loreyi* cold stress are unknown. Here, we compared the transcriptome data of the larvae, pupae, and adults of *M. loreyi* between cold stress and control treatments to explore the mechanisms by which this moth coped with cold tolerance. In this study, a total of 120.64 GB clean reads and 19,459 genes were observed in *M. loreyi’s* three developmental stages. Differences in gene expression patterns under the analysis of low-temperature acclimation revealed 1780 DEGs, with more upregulated genes than downregulated genes. This suggests that the positive regulation of these genes enhanced *M. loreyi’s* ability to withstand cold at various phases.

Previous studies indicated that insect responses to low-temperature stress are complex and require the involvement and control of several genes and pathways [26,29,36]. In this study, the enrichment analysis of the GO and KEGG pathway of these DEGs provided an apprehensible review and highlighted the biological mechanisms behind cold stress responses in three developmental stages of *M. loreyi*. GO annotation analysis showed that MF (48.07–51.89%) was predominant in this study, followed by BP (31.08–34.69%), which implied that most enzymes and proteins needed to be repaired after cold stress in *M. loreyi*. There were 10, 3, and 1 GO terms belonging to MF that were significantly enriched in larvae, pupae, and adults, respectively, while no GO terms showed a notable enrichment in the BP and CC categories. In the BP category, many GO terms related to the defense response, response to stress, or response to stimulus were simultaneously enriched in the top 10 in three stages of *M. loreyi*. In the CC category, non-membrane-bounded organelles were simultaneously enriched in the top 10 in larvae and adults. In addition, cofactor binding and coenzyme binding were simultaneously enriched in MF in larvae and pupae.

Significant differences in metabolism-related processes in *M. loreyi’s* three developmental stages exposed to cold stress were identified through KEGG analysis. In larvae, the majority of the cold-regulated DEGs showed significant enrichment in the following pathways, including the drug metabolism of other enzymes, peroxisome, biosynthesis of cofactors, pentose and glucuronate interconversion metabolism, arginine and proline metabolism, and the metabolism of xenobiotics by cytochrome P450, which is significantly different from that of *Spodoptera frugiperda* larvae exposed to cold stress [39]. The enrichment of the processing of proteins in the endoplasmic reticulum pathway and longevity regulating pathway of multiple species indicated that these pathways contributed to cold tolerance in *M. loreyi* pupae, and an identical phenomenon was observed in *S. frugiperda* larvae exposed to heat stress [39]. Additionally, many pathways related to energy metabolism were also involved in *M. loreyi* as a reaction to cold stress, such as lipid metabolism in larvae and pupae, starch and sucrose metabolism in pupae and adults, amino acid metabolism in larvae and adults, and amino sugar and nucleotide sugar metabolism in pupae. These findings demonstrated that there were significant differences in energy metabolism in three developmental stages of *M. loreyi* cold acclimation.

When insects are subjected to cold stress, the synthesis of most proteins related to metabolism declines [14,24]. GLD, catalyzing glucose into gluconolactone and regulating the activity of antioxidant enzymes, such as GST, SOD, and CAT, belongs to the oxidoreductase. In this study, the further identification of DEGs found that five genes encoding GLD were inhibited in *M. loreyi* larvae exposed to cold stress, indicating that these genes were more cold-sensitive than others, and the cold tolerance of *M. loreyi* larvae is mediated by GLD via metabolic pathways. A similar result was also found in *G. daurica* [24] and *L. sativae* [14].

In insects, environmental pressures had a significant impact on hormone biosynthesis, metabolism, and signaling, which in turn regulated insect tolerance to these stresses [39,40]. In this study, insect hormone biosynthesis pathways were enriched in pupae and adults but not in larvae. Previous research indicated that JHBP has a significant impact on the JH signaling pathway as a specific carrier and is sensitive to Destruxin A in silkworms [40,41], but little work has been conducted on its function in insect cold stress. In this study, *JHBP* was highly upregulated in larvae and pupae under cold stress but downregulated in adults. Our results indicated that JHBP may play a crucial part in the JH signaling pathway and is crucial for the response of *M. loreyi* to cold stress. In addition, JHE, an important enzyme in insect JH metabolism [42], was also significantly upregulated in *M. loreyi* larvae and adults after exposure to cold stress, and a similar result was also found in *S. frugiperda* larvae [39]. These results reflected the different responses of JHs involved in the different developmental stages of *M. loreyi* when exposed to cold stress.

FAS is an essential and multifunctional enzyme in the insect synthesis of fatty acids and lipid metabolism [43]. Previous studies showed that *FAS* was differentially expressed under insect cold stress, including *P. xylostella* [25], *G. daurica* [24], and *S. frugiperda* [44]. In this study, two *FAS* genes were highly downregulated in *M. loreyi* larvae and adults after exposure to cold stress. These results reflected the low metabolic rates in fatty acid metabolism in *M. loreyi* exposed to cold stress, and this may be a crucial tactic for this species to adapt to low temperatures.

Previous studies showed that *HSP* family genes are critical factors in insect response to low temperature and are usually upregulated in many insects, including *L. trifolii* [27], *Liriomyza sativae* [45], *Bradysia odoriphaga* [46], and *C. chinensis* [47]. In this study, the expression profiles of *HSPs* were analyzed after *M. loreyi* exposure to cold stress. In total, nine *HSP* genes were highly upregulated in the cold resistance of *M. loreyi* pupae, while no *HSP* gene was found upregulated in larvae and adults. Among these *HSP* DEGs, five *HSP70* genes were involved, which suggested that the induced HSP family exhibited a dominant pattern in both the quantity and expression level of *HSP70* genes, with HSP70 being the primary factor responsible for thermo-tolerance in *M. loreyi* pupae. This finding was consistent with the previous study in *Grapholita molesta* by Chen et al. [48] and by Li et al. [49] in *Monochamus alternates*. Moreover, two *HSP68*, one *HSP12.2*, and one *HSP83* gene were also involved in *M. loreyi* pupae after exposure to cold stress. These results imply that several *HSP* members may be in charge of cold stress in *M. loreyi* pupae.

In addition to HSPs, CPs were also involved in insects’ response to cold tolerance. Cuticles support body structure in insects, and CPs are recognized for having an important role in insect development and responses to environmental stress [50]. Cold-responsive cuticular genes have been reported in many insects including *Eogystia hippophaecolus* [51], *Micrarchus hystriculeus* [22], *L. trifolii* [52], *S. frugiperda* [44], and *Drosophila melanogaster* [53]. In this study, 25 cold tolerance-related genes encoding CPs were differentially expressed in three stages of *M. loreyi* cold resistance, including 14 genes in larvae, 11 genes in pupae, and 5 genes in adults. Among these DEGs, 22 genes were upregulated, suggesting that changes in the cuticle might be crucial to *M. loreyi’s* ability to withstand cold temperatures. A similar result was also found in *C. chinensis* [47].

Our research, in combination with other studies, indicated that the molecular mechanisms of insect responses to cold stress are complex regulatory processes and vary greatly between species and among different stages or populations of one species. The above findings indicated that there were notable variations in the molecular mechanisms in three developmental stages of *M. loreyi* exposed to cold stress, suggesting that different stages of this species may adopt different strategies to cope with cold tolerance. Furthermore, larvae and pupae, showing relatively higher cold tolerance than adults, are more responsive to cold stress at the molecular level and this may explain why this species overwinters as larvae and pupae. Our study helps to expand our knowledge of the ecological adaptation of *M. loreyi* to low temperatures and contributes to our understanding of adaptive evolutionary strategies in this and other species.

## Figures and Tables

**Table 1:** Data output quality and mapping rates for the examined samples of *M. loreyi*.

Sample	Raw Reads	Clean Reads	Clean Bases	Q20 (%)	Q30 (%)	GC (%)	Genome (%)
ML-Lck1	45861858	45081946	6.76 G	98.12	94.33	46.87	87.45
ML-Lck2	43440550	42760148	6.41 G	98.31	94.80	47.32	87.88
ML-Lck3	41811480	41257116	6.19 G	98.28	94.71	46.85	87.41
ML-Lt1	41850648	41309902	6.20 G	98.36	94.87	46.78	87.87
ML-Lt2	41701374	40990290	6.15 G	98.30	94.79	46.76	87.80
ML-Lt2	44947624	44250252	6.64 G	98.29	94.70	46.87	87.51
ML-Pck1	46227564	45271816	6.79 G	98.08	94.31	46.89	86.87
ML-Pck2	46664572	45687604	6.85 G	98.13	94.39	46.43	87.23
ML-Pck3	49603322	48836742	7.33 G	98.06	94.16	45.27	85.48
ML-Pt1	45422798	44217254	6.63 G	98.09	94.31	46.72	87.09
ML-Pt2	46297872	45608812	6.84 G	98.11	94.30	45.62	86.21
ML-Pt3	45883070	44873890	6.73 G	98.06	94.23	46.40	85.48
ML-Ack1	45339252	44392486	6.66 G	98.31	94.91	45.90	87.51
ML-Ack2	46758158	45718742	6.86 G	98.12	94.40	46.52	87.12
ML-Ack3	46380166	45431862	6.81 G	97.77	93.55	45.98	86.24
ML-At1	45297954	44457258	6.67 G	98.06	94.31	46.24	86.92
ML-At2	46342744	45417112	6.81 G	97.74	93.71	46.25	84.22
ML-At3	49549586	48729888	7.31 G	98.19	94.45	45.18	86.57

**Table 2:** Distribution of differential expressed genes (DEGs) in GO terms enriched in biological processes, cell components, and molecular functions.

GO Terms	Larvae (%)	Pupae (%)	Adults (%)
Biological process	229 (34.08)	115 (31.08)	68 (34.69)
Cellular component	120 (17.86)	63 (17.03)	32 (16.33)
Molecular function	323 (48.07)	192 (51.89)	96 (48.98)
Total	672 (77.33)	370 (71.15)	196 (55.84)

**Table 3:** Significantly enriched GO terms in molecular function in the DEGs by cold stresses.

Stage	GO Term	DEGs	Genes	*p*-Value	Padj
Larvae	cofactor binding	39 (12.07)	385 (6.26)	4.44 × 10^−5^	0.004542
	flavin adenine dinucleotide binding	17 (5.26)	110 (1.79)	5.10 × 10^−5^	0.004542
	coenzyme binding	22 (6.81)	179 (2.91)	0.000152	0.009061
	iron-sulfur cluster binding	9 (2.79)	42 (0.68)	0.000260	0.009244
	metal cluster binding	9 (2.79)	42 (0.68)	0.000260	0.009244
	oxidoreductase activity	43 (13.31)	489 (7.95)	0.000460	0.013652
	electron transfer activity	8 (2.48)	42 (0.68)	0.0013036	0.033148
Pupae	vitamin binding	7 (3.65)	34 (0.55)	6.83 × 10^−5^	0.011332
	pyridoxal phosphate binding	6 (3.13)	30 (0.49)	0.000272	0.015069
	vitamin B6 binding	6 (3.13)	30 (0.49)	0.000272	0.015069
Adults	odorant binding	7 (7.29)	83 (1.35)	0.000286	0.031746

**Table 4:** Top 20 statistics of pathway enrichment based on the DEGs.

Stage	Pathway	Number	Ratio	Padj
Larva	Drug metabolism—other enzymes	16	0.087	0.016
	Ribosome	17	0.092	0.016
	Porphyrin metabolism	10	0.054	0.018
	Caffeine metabolism	6	0.033	0.036
	Peroxisome	16	0.087	0.036
	Metabolism of xenobiotics by cytochrome P450	12	0.065	0.039
	Biosynthesis of cofactors	19	0.100	0.039
	Ascorbate and aldarate metabolism	9	0.049	0.049
	Drug metabolism—cytochrome P450	10	0.054	0.049
	Pentose and glucuronate interconversions	9	0.049	0.049
	Retinol metabolism	8	0.043	0.075
	Arginine and proline metabolism	6	0.033	0.122
	Nucleotide metabolism	9	0.049	0.298
	Proteasome	5	0.027	0.440
	Pyrimidine metabolism	6	0.033	0.520
	Cysteine and methionine metabolism	5	0.027	0.520
	Purine metabolism	10	0.054	0.520
	Base excision repair	4	0.022	0.520
	Sphingolipid metabolism	4	0.022	0.520
	Sulfur metabolism	2	0.011	0.520
Pupa	Longevity regulating pathway—multiple species	14	0.144	5.56 × 10^−7^
	Protein processing in endoplasmic reticulum	16	0.165	3.85 × 10^−5^
	Wnt signaling pathway	7	0.072	0.156
	Spliceosome	9	0.093	0.156
	Insect hormone biosynthesis	4	0.041	0.298
	Endocytosis	8	0.082	0.298
	Tyrosine metabolism	3	0.031	0.298
	Phenylalanine metabolism	2	0.021	0.474
	Sphingolipid metabolism	3	0.031	0.603
	Ubiquinone and other terpenoid-quinone biosynthesis	3	0.031	0.678
	MAPK signaling pathway—fly	5	0.052	0.678
	TGF-beta signaling pathway	3	0.031	0.678
	Caffeine metabolism	2	0.021	0.782
	mTOR signaling pathway	5	0.052	0.789
	Amino sugar and nucleotide sugar metabolism	3	0.031	0.789
	Phototransduction—fly	2	0.021	0.789
	Starch and sucrose metabolism	2	0.021	0.849
	Terpenoid backbone biosynthesis	2	0.021	0.849
	Hippo signaling pathway—fly	3	0.031	0.849
	Drug metabolism—other enzymes	5	0.052	0.849
Adult	Tyrosine metabolism	3	0.064	0.129
	Biosynthesis of cofactors	7	0.149	0.129
	Phenylalanine metabolism	2	0.043	0.129
	Ubiquinone and other terpenoid-quinone biosynthesis	3	0.064	0.129
	Histidine metabolism	2	0.043	0.129
	Lysine degradation	3	0.064	0.129
	Insect hormone biosynthesis	3	0.064	0.129
	Pyruvate metabolism	3	0.064	0.220
	Pantothenate and CoA biosynthesis	2	0.043	0.220
	Glycolysis/Gluconeogenesis	3	0.064	0.220
	Arachidonic acid metabolism	3	0.064	0.220
	Ascorbate and aldarate metabolism	3	0.064	0.220
	Motor proteins	4	0.085	0.220
	Circadian rhythm—fly	2	0.043	0.220
	Tryptophan metabolism	2	0.043	0.244
	Starch and sucrose metabolism	2	0.043	0.244
	Drug metabolism—cytochrome P450	3	0.064	0.250
	beta-Alanine metabolism	2	0.043	0.250
	Arginine and proline metabolism	2	0.043	0.250
	Peroxisome	4	0.085	0.250

## Data Availability

Data can be provided on request from the lead author.

## Supplementary Materials

The following supporting information can be downloaded at https://www.mdpi.com/article/10.3390/insects15070554/s1, Figure S1: Top 10 terms of GO enrichment analysis of the DEGs in biological process (BP), cellular component (CC), and molecular function (MF) in the larvae (A), pupae (B), and adults (C) of *M. loreyi* under cold stress. Table S1: The primers for RT-qPCR assay; Table S2: Sample and reference genome mapping statistics; Table S3: Expression levels of differentially expressed genes in *M. loreyi* larvae between cold stress and control group; Table S4: Expression levels of differentially expressed genes in *M. loreyi* pupae between cold stress and control group; Table S5: Expression levels of differentially expressed genes in *M. loreyi* adults between cold stress and control group.
