# Supplementary material for: Comparative Transcriptome Analysis Reveals Different Responses in Three Developmental Stages of Mythimna loreyi to Cold Stress
Source: Insects. 2024 Jul 22;15(7):554. doi: 10.3390/insects15070554 (PMC11276649; doi:10.3390/insects15070554)
Supplement: Supplementary file 1 [file insects-15-00554-s001.zip › Figures-S1.pdf]

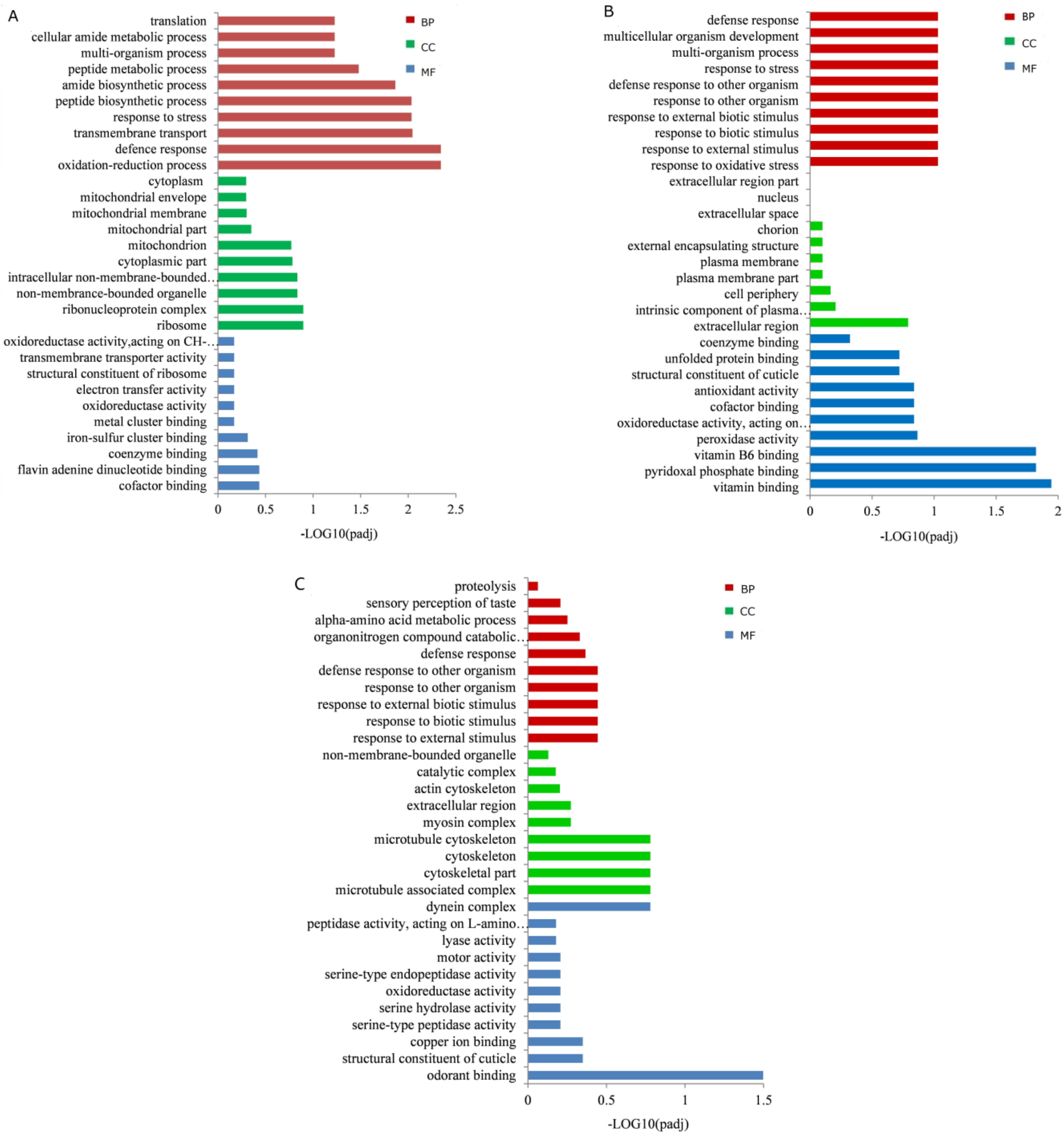

Figure S1. Top 10 terms of GO enrichment analysis of the DEGs in biological process (BP), cellular component (CC) and molecular function (MF) in the larvae (A), pupae (B) and adults (C) of *M. loreyi* under cold stress. The x-axis indicates the three main categories. The y-axis indicates the degree of GO enrichment. Red bars indicate GO terms in BP category. Green bars indicate GO terms in CC category. Blue bars indicate GO terms in MF category.
